# Real-time deep learning-based image guiding and automated left ventricular measurements to reduce test-retest variability

**DOI:** 10.1136/openhrt-2025-003783

**Published:** 2025-12-07

**Authors:** Håkon Pettersen, Sigbjorn Sabo, David Pasdeloup, Erik Smistad, Sindre Olaisen, Andreas Østvik, Stian Stølen, Bjørnar Leangen Grenne, Lasse Løvstakken, Havard Dalen, Espen Holte

**Affiliations:** 1 Department of Circulation and Medical Imaging, Norwegian University of Science and Technology, Trondheim, Trøndelag, Norway; 2 Clinic of Cardiology, St Olav’s Hospital HF, Trondheim, Trøndelag, Norway; 3 Department of Internal Medicine, Levanger Hospital, Levanger, Nord-Trondelag, Norway; 4 Department of Health Research, SINTEF Digital, Trondheim, Trondheim, Norway; 5 Clinic of Cardiology, St Olav's Hospital University Hospital in Trondheim, Trondheim, Norway

**Keywords:** Echocardiography, Diagnostic Imaging, Heart Failure

## Abstract

**Aims:**

To evaluate the effect of combining real-time deep learning (DL)-based guiding and automated measurements of left ventricular (LV) volumetric measurements and strain.

**Methods and results:**

Patients (n=47) with mixed cardiac pathology were examined by two sonographers and one reference cardiologist. A real-time DL guiding tool to avoid LV foreshortening was used by one sonographer only per patient. Automated DL-based measurements from the sonographer using the guiding tool were paired with automated measurements from the reference cardiologist (artificial intelligence (AI)-assisted echocardiography), while manual measurements from the sonographer not using the guiding tool were paired with manual measurements from the reference cardiologist (standard echocardiography). The variability of LV EDV, LV ESV, ejection fraction (LV EF) and global longitudinal strain (LV GLS) was compared for standard echocardiography versus AI-assisted echocardiography. Coefficients of variation were lower for AI-assisted echocardiography compared with standard echocardiography (6% vs 15% for LV EDV (p<0.001), 10% vs 19% for ESV (p<0.001) and 7% vs 11% for GLS (p=0.047), respectively). For LV EF, the coefficients of variation were similar across groups (8% vs 9%, p=0.503, respectively). In exploratory analyses, automated measurements alone (all p≤0.002) but not the guiding tool (all ≥0.199) explained the improved variability for LV EDV, ESV and GLS.

**Conclusions:**

AI-assisted echocardiography combining DL-based real-time guiding and automated measurements significantly reduced the variability of LV EDV, ESV and GLS when compared to standard echocardiography. Among experienced operators, automated measurements were more beneficial than real-time guiding.

**Trial registration number:**

ClinicalTrials.gov, ID: NCT04580095.

**WHAT IS ALREADY KNOWN ON THIS TOPIC**

- Echocardiographic measurements of left ventricular (LV) volumes, ejection fraction and global longitudinal strain (GLS) have a significant test-retest variability. LV apical foreshortening, together with manual measurements, is a significant contributor to the variability between recordings. We aimed to use artificial intelligence (AI) to compare the use of real-time operator guidance to reduce LV foreshortening and automated measurements with respect to test-retest variability among experienced operators.

**WHAT THIS STUDY ADDS**

- AI-assisted echocardiography, with real-time operator guidance to reduce LV apical foreshortening, combined with automated measurements, significantly reduced variability of LV end-diastolic volume, end-systolic volume and GLS, when used by highly experienced sonographers compared with standard echocardiography.

**HOW THIS STUDY MIGHT AFFECT RESEARCH, PRACTICE OR POLICY**

- Further studies in larger populations and with a wider span of operators are needed to reveal the clinical impact of implementing AI in echocardiography.

## Introduction

 Optimising echocardiographic acquisitions and measurements according to recommendations may be challenging even for experienced operators.^1^,^4^ The importance of standardising image acquisition is highlighted in current guidelines as it influences the quality provided by echocardiography laboratories worldwide.^5 6^

Key parameters for the evaluation of left ventricular (LV) size and function include end-diastolic volume (LV EDV), end-systolic volume (LV ESV), ejection fraction (LV EF) and global longitudinal strain (LV GLS). However, these measurements are both time-consuming and hampered by a significant test-retest variability.^47^,^9^ Moreover, LV apical foreshortening is a significant contributor to the variability between recordings.^10^

**Figure 1:**
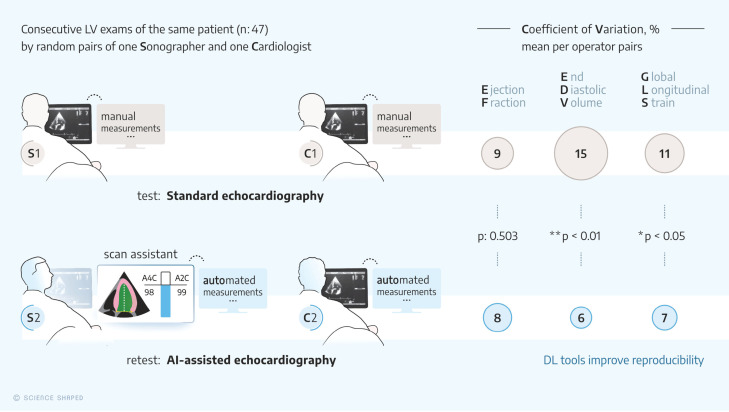
Graphical abstract: Upper panel: The standard echocardiography group with one of three sonographers (S1) and one of four cardiologists (C1) not supported by any DL tool. Bottom panel: The AI-assisted echocardiography group with another sonographer (S2) using DL-based guiding to avoid left ventricular foreshortening combined with automated measurements and automated measurements from the same cardiologist’s recordings as included in the standard echocardiography group (C2). Right panel: Compared to the standard echocardiography group, the coefficients of variation were lower for the AI-assisted echocardiography group for measurements of left ventricular volumes and global longitudinal strain, but not for ejection fraction.

The potential of artificial intelligence (AI) in echocardiography has recently gained considerable attention.^11 12^ While AI, and in particular deep learning (DL), may improve image acquisition and automatise relevant measurements,^813^,^20^ most studies have focused on the use of DL for automatic measurements retrospectively.^15^,^20^ Real-time guiding has shown the potential to support inexperienced operators in acquiring adequate echocardiographic views.^21 22^ We have recently shown that DL guiding with real-time feedback of LV length and foreshortening was helpful in reducing LV apical foreshortening even in experienced operators.^8^ However, this did not translate to reduced variation when combined with manual measurements.^8^ Our group has also developed and validated DL tools to optimise recordings and analyses of LV volumes, LV EF and LV GLS.^23^,^25^ The aim of the current study was to evaluate whether the combination of real-time feedback of LV length using a DL software during echocardiographic scanning and automated analyses of LV volume and function could improve the agreement and reliability of the LV measurements compared with standard echocardiographic procedures. Subsequently, we aimed to compare the contributions of real-time operator guidance to reduce LV foreshortening and automated measurements with respect to test-retest variability among the experienced operators (figure 1).

## Methods

### Population and study design

Patients scheduled for echocardiography at the echocardiography laboratory at St. Olav’s University hospital were recruited. The only inclusion criteria were an indication for comprehensive echocardiography and willingness to provide informed consent. Patients with atrial fibrillation or an indication for contrast echocardiography were excluded. The study was approved by the regional committee of medical research ethics (identification number 7160) and performed according to the Helsinki declaration. All participants freely provided informed written consent prior to inclusion.

Each participant underwent three consecutive echocardiographic examinations in left lateral decubitus position by three different operators without leaving the examination bench. The time delay was minimised just to the change of operators. The participants remained in a supine position between examinations to minimise physiological influence on the variability between the exams. The first and second examinations were performed by two (of three) randomised sonographers and the third exam by one (of four) randomised cardiologist. All personnel were affiliated with the European Association of Cardiovascular Imaging (EACVI) accredited echocardiography laboratory at St. Olav’s Hospital, Trondheim, Norway. A Vivid E95 scanner (version 202, GE HealthCare, Horten, Norway) with a phased array transducer (4Vc, GE HealthCare) was used. The current study was part of a larger initiative, and we have previously published the effect of real-time guidance during scanning to optimise image acquisitions.^8 26^

Prior to data collection, the sonographers were introduced to the real-time DL tool and trained on 10 patients each. The three sonographers were randomly allocated to the role as being sonographer with or without using the DL guiding tool and automated measurements. The first examination was comprehensive and aligned to the American Society of Echocardiography and EACVI recommendations.^5 6^ The other examinations focused on study-specific recordings and analyses. All echocardiograms included the standard apical two-chamber (A2C), four-chamber (A4C) and long-axis views. Moreover, the cardiologists’ exams also included a triplane LV recording for reference measurements of LV length. All study-specific recordings were deidentified before data analyses and organised in similar datasets to ensure blinding.

Manual measurements of LV volumes, LV EF and GLS by the sonographer who recorded images without the DL guiding tool paired with the corresponding manual measurements by the cardiologist comprised the *standard echocardiography* group. Similarly, automated measurements from the sonographer who recorded images using the DL guiding tool paired with the corresponding automated measurements from the same cardiologist comprised the *AI-assisted echocardiography* group. Manual measurements should be representative for the three recorded cycles, while the average of the three recorded cycles was used for automated measurements.

### Details of the real-time DL foreshortening detection algorithm

**Figure 2:**
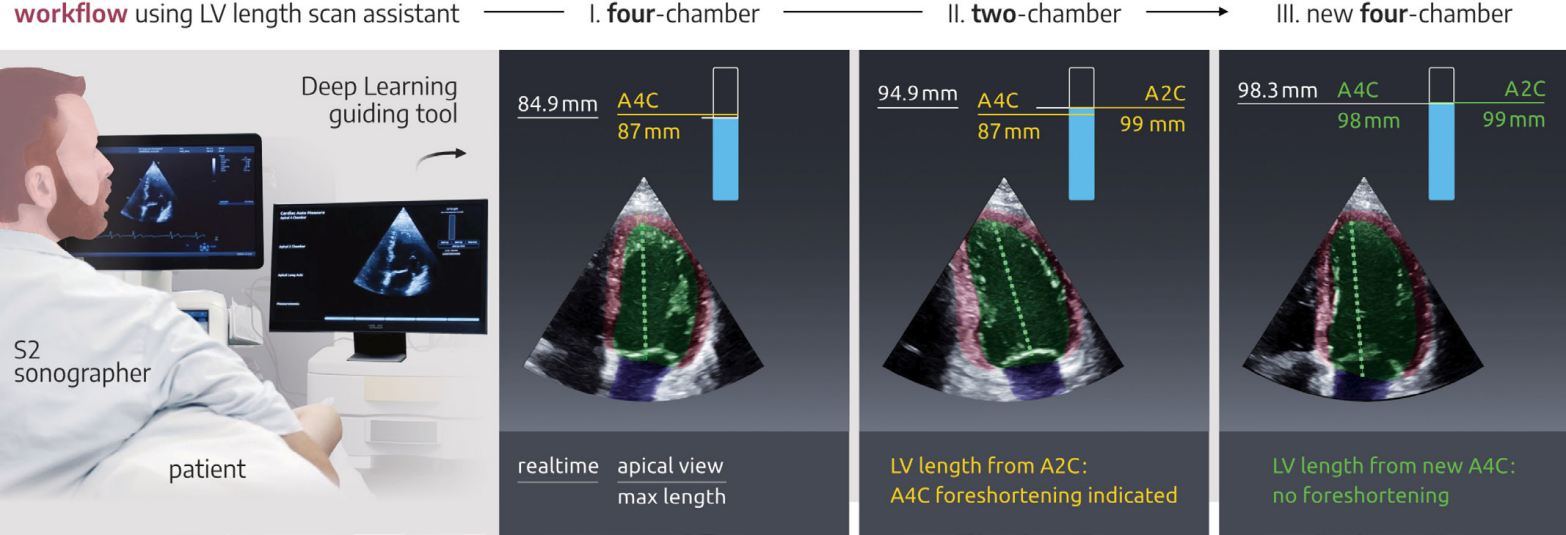
Real-time deep learning-based operator guiding. Left panel: the setup for real-time deep learning-based guiding of sonographers during echocardiographic scanning where the ultrasound scanner monitor shows the original ultrasound recording (left monitor) and the guiding tool is shown on the right monitor by real-time streaming of data. The right panel shows three examples of ultrasound images with real-time feedback from the guiding tool. The first image shows an apical four-chamber (A4C) recording with left ventricular (LV) length of 87 mm. The second image shows that the apical two-chamber (A2C) recording has a LV length of 99 mm indicating foreshortening of the first A4C recording. The last image shows an optimised A4C recording where the DL guidance tool indicates no foreshortening.

The DL guiding tool provides real-time feedback to the user about view-specific LV length and whether any of the three specified apical views may be foreshortened. Details of the training and performance of the DL algorithm have been previously published.^8 23^ Shortly, the timing network was trained on 500 patients with A4C/A2C 2D-recordings from the public available CAMUS dataset. View classification network was trained on 500 patients with multiple views with manual labelling. The LV foreshortening algorithm was trained using publicly available CETUS dataset. From this 3D dataset, 2D views were constructed by systematically slicing new planes with predefined off-sets from the manually labelled apical and basal points. Separate DL networks automatically performed cardiac view classification, cardiac phase detection (systole/diastole) and LV segmentation. The LV length was calculated based on apical and basal LV landmarks extracted from the segmentation at ED and presented for comparison between the three standard apical views. Additionally, a warning sign occurred during excessive apical movement in each chamber view using a cut-off of 3 mm, respectively. Furthermore, LV length differences <3 mm between the three apical views were accepted by the software and indicated with green coloured numbers on the feedback output. If the difference in LV length across views exceeded 3 mm, the view specific foreshortening was indicated by change from green to yellow colour. During scanning, the operator could redo the recordings in case LV foreshortening of any view was detected. The workflow during scanning was as followed: the operators acquired LV recordings in the following order – A4C, A2C, ALAX. During real-time scanning the operator feedback was shown together with the actual ultrasound recording on a separate monitor beside the ultrasound scanner connected through an Ethernet cable (figure 2 and online supplemental video 1). For each of the views the operator received feedback in real-time of the length of the LV and apical foreshortening exceeding 3 mm. Except for the first view the view-specific LV length was compared to other views. In case of foreshortened views, the operator could replace the foreshortened recording.

### Analyses of LV size and function

*Manual measurements* were done retrospectively blinded to the other users, using EchoPAC software only (V.204; GE HealthCare, Horten, Norway). Each operator independently analysed their recordings blinded to the other operators’ or automated measurements, regardless of the inclusion of automated analyses in the primary outcomes. The LV endocardial border was traced in A4C and A2C at ED and end-systole and the manual measurements of LV EDV, LV ESV and LV EF were calculated from the traces using the method of discs’ summation. LV GLS was measured using the automated functional imaging (AFI) package in with an 18-segment model. To better account for the operators’ decision whether to approve or discard segmental values, we also calculated GLS using a 16-segment model based on the segmental values.^6^

*Automated DL measurements of LV volumes, EF and GLS* were done retrospectively using two separate DL tools independent of group allocation. Details of the DL algorithms measuring LV volumes, EF^9^ and LV GLS^24 27^ have been previously published. First, four separate DL networks performed cardiac view classification, timing of cardiac events, segmentation of the LV lumen and myocardium and tracking of the myocardium. All parts of the automated analyses were overseen by experienced cardiologists and measurements were performed in all available cardiac cycles. In short, the LV EDV and ESV were estimated using the method of disc summation based on automatic segmentation of the endocardial border at ED and end-systole in A4C and A2C views and LV EF was calculated. Second, a DL network tracked the LV myocardial motion for measurements of LV GLS based on centreline extraction from the segmented LV myocardium. Vie-specific measurements of LV GLS were calculated as the percentage change of the centreline length.

*The reference measurements of LV length* methodology have been recently published.^8^ In short, view-specific LV length was measured by two operators in all standard apical recordings at ED. Reference measurements of LV length were similarly measured in the cardiologists’ tri-plane recordings. The view-specific reference LV length minus the operators’ view-specific LV length measured from the 2D recordings was used in the analyses of LV foreshortening.

### Statistics

As the data were normally distributed, continuous variables are presented as mean±SD. Paired sample t-tests were used for comparison across the pairs of operators. The coefficient of variation (CoV) was calculated within pairs of operators. The primary aim was evaluated between the standard echocardiography group and the AI-assisted echocardiography group. The secondary aims were evaluated in the full dataset according to the use of the DL guiding tool and mode of measurements. Multivariable linear regression analyses were used to evaluate the associations of within pairs of CoV with use of the DL guiding tool and modes of measurements. The LV GLS data presented in tables and figures were derived from the 16-segment model, with patients excluded if they had fewer than 12 approved segmental values. There was no difference in the results whether the 16-segment model or AFI with 18-segment model was used. Inter-rater reliability was calculated using intraclass correlations (ICCs) with a two-way random model defined by absolute agreement. Intrarater (beat-to-beat) ICCs were calculated using a two-way mixed-effect model defined by absolute agreement in the dataset of single measurements. ICC values<0.5 were considered poor, 0.5–0.75 moderate, 0.75–0.9 good and >0.9 excellent. Bootstrapping of 1000 samples was used to estimate the 95% CI. All statistical analyses were done using SPSS Statistics (V.29.0.1.0; IBM, Armonk, New York, USA).

## Results

### Population

The demographic characteristics of the participants are summarised in table 1. A total of 56 patients were recruited. Three patients were excluded due to non-sinus rhythm and six were excluded due to need for contrast echocardiography. Thus, a total of 47 patients were included in the analyses. Among these, 46% were women, and the mean (SD) age was 63 (15) years. LV function and heart morphology varied substantially across patients with approximately one-third having reduced LV function and about 20% having either LV hypertrophy, known myocardial infarction or at least moderate valvular disease.

**Table 1:** Baseline characteristics of the study population

Variable	Mean (SD) or number (%)
Age in years	63 (15)
Women	21 (46%)
*Clinical characteristics*	
LV EF<50%	15 (32%)
LV hypertrophy	11 (23%)
LV dilatation*	7 (15%)
Acute myocardial infarction	8 (17%)
Moderate or severe valvular disease	10 (21%)
Chronic obstructive pulmonary disease	1 (2%)
Hypertension	11 (23%)
Body mass index, kg/m²	27 (6)
Heart rate, beats per minute	69 (11)
Systolic blood pressure, mm Hg	140 (21)
*Echocardiographic characteristics* †	
LV EDV, biplane, mL	127 (56)
LV ESV, biplane, mL	65 (50)
LV EF, biplane, %	53 (12)
LV GLS, absolute %	16 (5)

Values are mean (SD) or number (%).

* Defined as diameter >5.8 cm (men) and >5.3 cm (women).

† As measured by reference cardiologist.

EDV, end-diastolic volume; EF, ejection fraction; ESV, end-systolic volume; GLS, global longitudinal strain; LV, left ventricular.

### Real-time DL intervention and measurement variability

Table 2 and the graphical abstract show that the agreement between operators in the AI-assisted echocardiography group was significantly better than in the standard echocardiography group for measurements of LV EDV (mean CoV 6% vs 15%, p<0.001), LV ESV (mean CoV 10% vs 19%, p<0.001) and LV GLS (mean CoV 7%-points vs 11%-points, p=0.047). The agreement of LV EF did not differ between groups (p=0.503). Sonographers using DL-based guiding foreshortened the LV 0.5 (2.5) mm compared with triplane reference measurements, while sonographers without guiding foreshortened the LV 2.1 (2.6) mm (p=0.08), respectively. The difference in mean =1.6 mm was significant. For the cardiologists, the corresponding foreshortening was 0.2±(1.7) mm, with a significant difference to the standard echocardiography sonographer. The latter data have been recently published, but are included here for the completeness of the presentation.^8^ As evaluated by humans, overseeing the DL algorithm correctly identified the views and performed retrospective automatic measurements of LV volumes, LV EF and LV GLS in all patients and views (data not shown).

**Table 2:** Agreement of left ventricular measurements according to operator groups

	Standard echocardiography*	AI-assisted echocardiography†	P value
LV EDV, CoV in %	15 (10)	6 (6)	<0.001
LV ESV, CoV in %	19 (14)	10 (9)	<0.001
LV EF, CoV in %-points	9 (7)	8 (8)	0.503
LV GLS, CoV in %-points	11 (9)	7 (6)	0.047

Otherwise, as in table 1.

* No real-time DL-based guiding combined with manual analyses.

† Real-time DL-based guiding of sonographers combined with automated analyses.

CoV, coefficient of variation; DL, deep learning; EDV, end-diastolic volume; EF, ejection fraction; ESV, end-systolic volume; GLS, global longitudinal strain; LV, left ventricular.

### Significance of the two elements of the DL intervention

**Figure 3:**
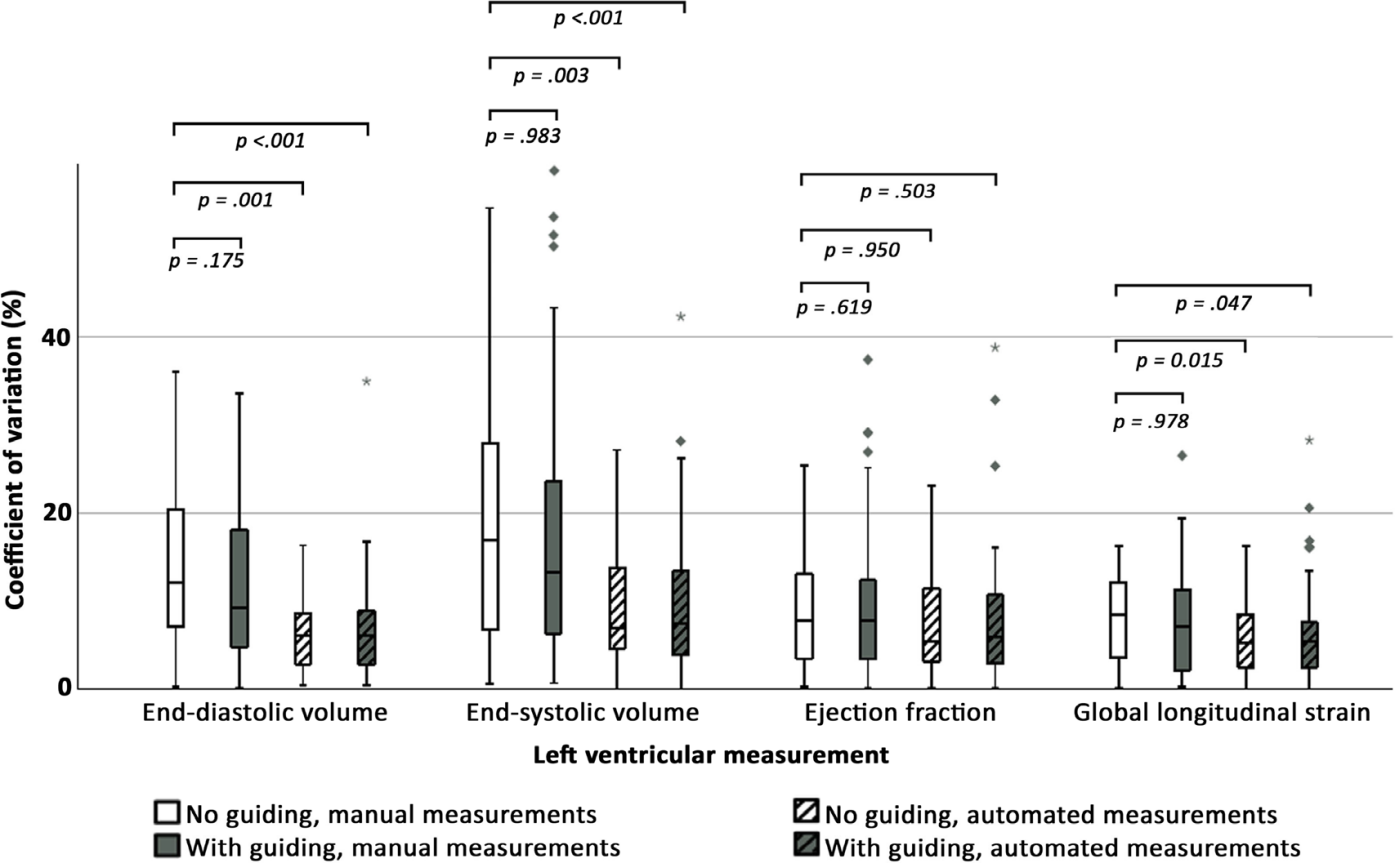
Coefficients of variation for left ventricular measurements according to groups and measurement methods. Box plots showing within pairs coefficients of variation. P values compared to standard echocardiography (no real-time guiding with manual measurements) are shown on top. Coefficients of variation for left ventricular ejection fraction and global longitudinal strain are presented in %-points.

The full details of LV size and function measurements according to the use of the DL guiding tool and mode of measurements are summarised in figure 3 and table 3. Compared with manual standard echocardiography, automated measurements showed lower variability for LV EDV, ESV and GLS, regardless of DL guiding tool use (all p≤0.047). Adjusted regression found that only automated measurements (all p≤0.002), not DL guiding (all p≥0.199), were significantly closer to cardiologists’ reference values (data not shown). Manual cardiologists’ reference measurements of LV EDV were larger than automated measurement and manual measurements by sonographers, with mean differences of 7–13 mL and 15–18 mL, respectively (all p≤0.007). For LV ESV, the cardiologists’ reference measurements showed no meaningful difference to automatic measurements with mean differences of 1–4 mL (all p≥0.209) but were significantly larger than manual measurements by sonographers with mean differences of 9 mL (all p≤0.002). For LV EF, the differences were small, with no significant differences across operators. Correspondingly, automated LV EF measurements were 2–6%-points lower than manual measurements across operators, with the smallest differences for cardiologists (all p≤0.039). Similarly, LV GLS showed no clinically significant differences across groups (all p≥0.275), but automated measurements were 1%-point lower for all operators.

**Table 3:** Measurements of LV size and function according to methodology

	Manual measurements	Automated DL measurements	P value
*Left ventricular end-diastolic volume, mL*			
Sonographer without guiding	112 (54)*	120 (47)	0.050
Sonographer with guiding	109 (53)*	114 (47)†	0.450
Cardiologist (reference)	127 (56)	117 (46)	0.002
*Left ventricular end-systolic volume, mL*			
Sonographer without guiding	56 (49)*	64 (33)†	0.028
Sonographer with guiding	56 (47)*	61 (37)	0.053
Cardiologist (reference)	65 (50)	60 (35)	0.176
*Left ventricular ejection fraction, %-points*			
Sonographer without guiding	53 (14)	48 (10)†	0.005
Sonographer with guiding	55 (13)†	49 (10)	<0.001
Cardiologist (reference)	53 (12)	51 (9)	0.039
*Left ventricular global longitudinal strain, absolute %*			
Sonographer without guiding	16 (5)*	15 (4)	0.503
Sonographer with guiding	16 (5)*	15 (4)	0.414
Cardiologist (reference)	16 (5)	15 (4)	0.275

The manual measurements were recently published^8^ but are included to ease the interpretation. P values indicate level of significance for differences between manual and automatic measurements

* Differences from cardiologists’ measurements by same method: p<0.05

† p 0.05–0.10, not annotated when p>0.10

DL, deep learning; LV, left ventricular.

As shown by the inter-rater ICCs in table 4, reliability was good or excellent for automated measurements of LV volumes, EF and GLS with no significant effect of the real-time guiding tool (all ICCs ≥0.77). Moreover, intrarater beat-to-beat for automated measurements of the same LV indices showed excellent reliability with all ICCs >0.95 (data not shown).

**Table 4:** Inter-rater reliability of automated measurements according to operator pairs

	Standard echocardiography*	AI-assisted echocardiography†	P value
LV EDV, CoV in %	8 (8)	6 (6)	0.233
LV ESV, CoV in %	11 (9)	10 (9)	0.592
LV EF, CoV in %-points	9 (9)	8 (8)	0.389
LV GLS, CoV in %-points	6 (4)	7 (6)	0.184
LV EDV, ICC (95% CI)	0.96 (0.92 to 0.98)	0.98 (0.96 to 0.99)	–
LV ESV, ICC (95% Cl)	0.96 (0.93 to 0.98)	0.98 (0.96 to 0.99)	–
LV EF, ICC (95% CI)	0.77 (0.58 to 0.87)	0.82 (0.68 to 0.90)	–
LV GLS, ICC (95% CI)	0.97 (0.94 to 0.98)	0.94 (0.89 to 0.97)	–

* Retrospective AI-based measurements from the standard echocardiography group.

† Real-time DL-based guiding of sonographers combined with automated analyses.

AI, artificial intelligence; CoV, coefficient of variation; DL, deep learning; EDV, end-diastolic volume; EF, ejection fraction; ESV, end-systolic volume; GLS, global longitudinal strain; ICC, intraclass correlation; LV, left ventricular.

## Discussion

In this study evaluating the effect of AI-assisted echocardiography combining real-time foreshortening guidance during echocardiography scanning with automated DL-based measurements on the variability of LV measurements, the main findings were: a 36–60% relatively lower CoV for LV EDV, LV ESV and GLS. Second, no significant difference for LV EF compared with standard echocardiography. Lastly, automated DL-based measurements were the main contributor to the reduced variability of LV EDV, ESV and GLS, while the DL guiding tool added no significant benefit among the experienced operators. This adds to the knowledge base on the clinical usefulness of real-time image analysis based on DL for image standardisation and automated measurements and highlights the power of automated image analyses to reduce the user dependency of echocardiography.

### Population

As the study participants were recruited from in-hospital wards and the outpatient clinic based on referral for echocardiography and the only exclusion criteria were the presence of arrhythmia or the need for contrast enhanced imaging, we expect that the results may be generalised broadly. As shown by the presented results, comorbidities such as previous myocardial infarction and hypertension, and LV dysfunction were common, and included participants were distributed across the whole span of LV EF ranging from 17% to 76% by the reference cardiologists’ manual measurements. Both the real-time guidance tool and the automated measurement algorithms showed a feasibility of 100% among the experienced operators. With respect to the generalisation of the results, caution relates to patients with deviations from normal morphology as they were underrepresented in the present study as well as the training material.^19 23^ Moreover, as the operators were highly experienced, future studies including larger datasets and operators with varying experience are needed for evaluation of the clinical impact of improving standardisation and measurement variability.

### Real-time guiding and mode of measurements

The improved agreement for the AI-assisted echocardiography group compared with standard echocardiography is clinically important and indicates a significant potential to improve standardisation and reduce variability even in experienced operators. Moreover, the finding of no significant differences in LV EF between groups could partly be explained by LV EF being the difference of end-diastolic and ESVs divided by the EDV. Thus, reducing variability for the volumetric measurements does not directly translate to improved variability for LV EF. The limited population size may also have reduced the power to detect differences across groups for LV EF measurements.

When automated measurements replaced manual measurements in the standard echocardiography group, no significant difference between groups was present, despite sonographers in the AI-assisted echocardiography groups having less foreshortening compared with the sonographers without guiding. This indicates that the difference of 1.6 mm in LV foreshortening across sonographers with or without guiding was less important than the variability introduced from the manual measurement procedures. This finding should not be interpreted as an unexpected finding when considering the variability in test-retest scenarios from previous studies.^2 4 9 24^ It is not known if this finding would be consistent across less experienced operators, even though it would be expected that the impact of the DL guiding tool would be higher for inexperienced users.^21 22^ As only exact numbers provided by the calculations of LV EF were used, the findings were not influenced by digit bias.^28^ Also, the fact that automated measurements were averaged over three cardiac cycles compared with a single measurement representative for the three available cardiac cycles in the standard echocardiography group may reduce the variability by producing more robust measurements. However, as automated measurements showed excellent beat-to-beat reliability across operators, it is not expected that this was the reason behind the presented results. Importantly, even small differences in LV EF or LV GLS may influence treatment and follow-up of heart failure patients,^28 29^ and thus, it is important to minimise the variability of functional LV measurements.

### The two elements of the DL intervention

This study showed that the automated measurements, not the DL guiding tool, were an important contributor to reducing the variability between experienced echocardiographic operators. To the best of our knowledge, this has not been evaluated in previous studies. As shown by the reduction of LV foreshortening from 2.1 mm to 0.5 mm using the DL guiding tool, this study showed that real-time guiding may improve image recordings even among experienced operators, even though the impact was too small to improve the test-retest variability. Aligned to other studies, we present data supporting that AI-based image analyses for guiding of recordings and automated measurements could increase the diagnostic yield of echocardiography to improve care for large groups of patients.

Automated measurements of LV volumes, EF and GLS were somewhat lower than manual measurements. It is well known that such differences may originate from systemically different endocardial border delineation and segmentation of the LV myocardium, variable quality of tracking, learning-based regression to the mean for segmentation and tracking and systematic differences in timing.^23 27^ It may be expected that the first three elements may have been of importance for the shown differences in LV volume measurements. For the presented GLS differences, it is more probable that the difference relates to the fact that the automated methods calculate GLS based on the centreline shortening while the manual method is based on tracking of the whole myocardial wall. From a clinical point of view, real-time guiding combined with automatic measurements of LV systolic function could also have the potential to reduce the workload and increase the proportion of adequately quantified recordings by eliminating the need for time-consuming analyses.^9^

### Strengths and limitations

The main strengths of the study relate to: (1) all echocardiographic examinations were performed by the clinically relevant group of experienced operators at a European Association of Cardiovascular Imaging accredited echocardiography laboratory, (2) the prospective study design evaluating outcomes of test-retest variability of measurements when real-time guidance and automated measurements were introduced compared with conventional clinical workflow, (3) the robust and validated DL-based guiding tool able to run in real-time keeping the human-in-loop and (4) the wide span of heart morphology and systolic function of the study population, ranging from young and healthy patients with good acoustic windows to elderly patients with severe heart failure as well as patients with poor image quality.

This study also has important limitations. First, the sample size was based on calculations for the primary endpoint. Thus, the relatively low number of patients limits the power for exploratory analyses. Inclusion of more study participants would have increased the power for evaluating the influence of AI-assisted echocardiography for the agreement of LV EF and for comparisons of DL guiding vs automated measurements.

## Conclusions

AI-assisted echocardiography with real-time operator guidance to reduce LV apical foreshortening combined with automated measurements significantly reduced variability of LV EDV, ESV and GLS when used by highly experienced sonographers compared with standard echocardiography. Even though this did not translate into reduced variability for LV EF measurements, the use of automated DL image analysis has the potential to improve echocardiographic precision and the proportion of well-quantified echocardiograms. Similarly, it can reduce the workload and time consumption of echocardiography. Further studies in larger populations and with a wider span of operators are needed to reveal the clinical impact of implementing DL in echocardiography.

## Supplementary material

10.1136/openhrt-2025-003783 online supplemental file 1

10.1136/openhrt-2025-003783 online supplemental file 2

## Data Availability

Data are available upon reasonable request.
