# Supplementary material for: Real-time deep learning-based image guiding and automated left ventricular measurements to reduce test-retest variability
Source: Open Heart. 2025 Dec 7;12(2):e003783. doi: 10.1136/openhrt-2025-003783 (PMC12699943; doi:10.1136/openhrt-2025-003783)
Supplement: online supplemental file 1 [file openhrt-12-2-s001.pdf]

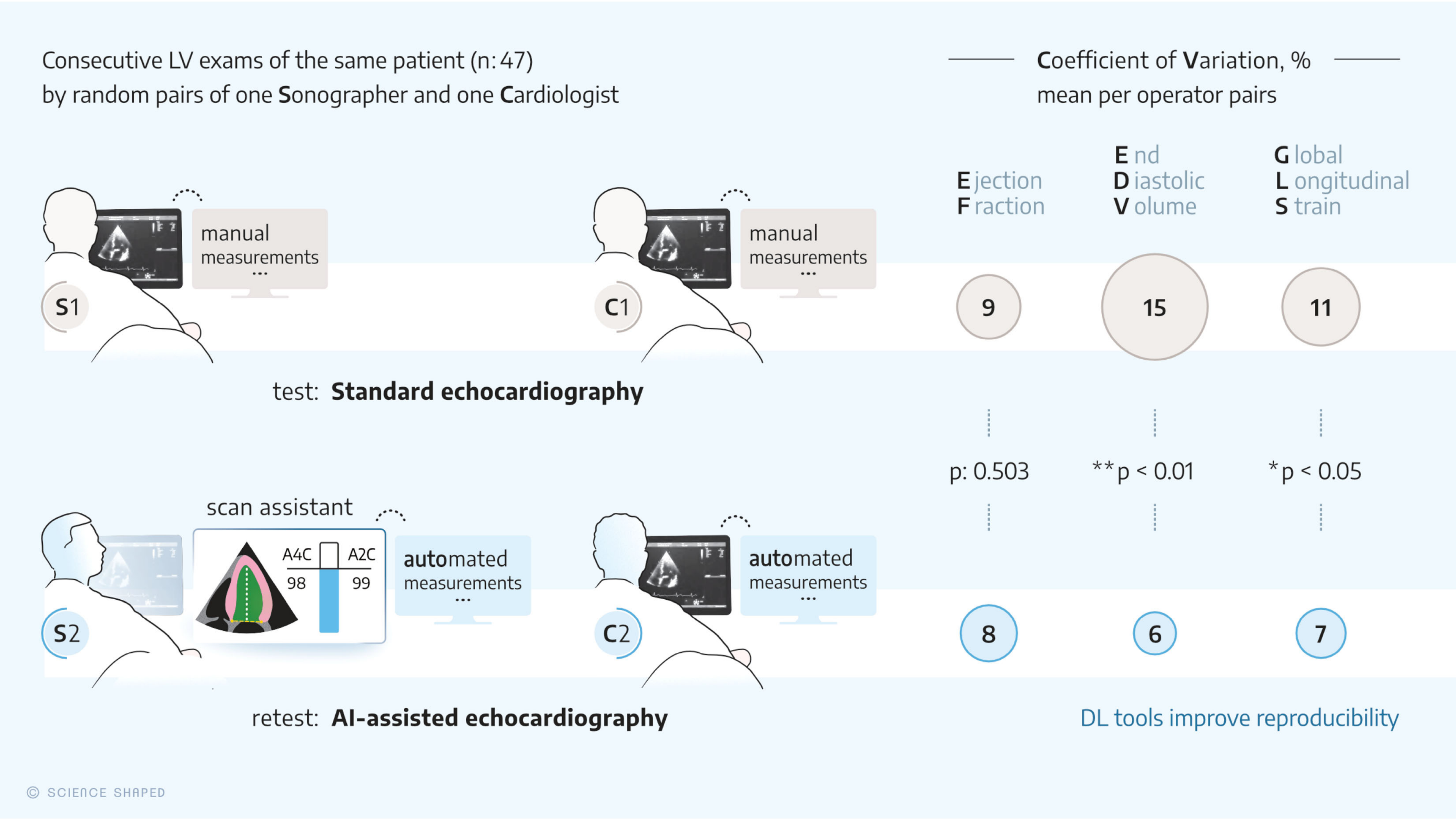
Consecutive LV exams of the same patient (n: 47)
by random pairs of one Sonographer and one Cardiologist
Coefficient of Variation, %
mean per operator pairs
Ejection Fraction
End Diastolic Volume
Global Longitudinal Strain
S1
manual measurements ...
C1
manual measurements ...
9
15
11
test: Standard echocardiography
p: 0.503
** p < 0.01
* p < 0.05
scan assistant
A4C
98
A2C
99
S2
automated measurements ...
C2
automated measurements ...
8
6
7
retest: AI-assisted echocardiography
DL tools improve reproducibility
© SCIENCE SHAPED
